# Policies and Practices Catalyzing the Use of Generic Medicines: A Systematic Search and Review

**DOI:** 10.4314/ejhs.v31i1.19

**Published:** 2021-01

**Authors:** Shahmoradi Mostafa, Mosadeghrad Ali Mohammad, Jaafaripooyan Ebrahim

**Affiliations:** 1 Ph.D. Candidate, Department of Health Management and Economics, School of Public Health, Tehran University of Medical Sciences, Tehran, Iran; 2 Associate Professor, Department of Health Management and Economics, School of Public Health, Tehran University of Medical Sciences, Tehran, Iran

**Keywords:** Generic medicines, Policies and practices, Brandgeneric, Cost saving, Systematic review

## Abstract

**Background:**

The use of generic medicines instead of branded, is one of the main policies to decrease the expenditures and provide access to affordable and essential medicines in low and middle-income countries. The present study aims to systematically create a comprehensive synthesis of demand-side policies, encouraging the use of generic medicines.

**Methods:**

The study systematically searched and reviewed the articles in Medline, Scopus, Web of Science, and Embase from 1.1.2000 to 12.5.2019. A total of 6435 records were identified during this period of time (Medline (n=315), Scopus (n=4323), Web of Science (n=71) and Embase (n=1726)). All stages are conducted according to the Preferred Reporting Item for Systematic Reviews and Meta-Analyzed (PRISMA).

**Results:**

The encouraging policies and practices were classified into four categories from 44 articles analyzed, including; Prescribing, Dispensing, Patients/consumers, and healthcare organizations. Subthemes were also explored in relation to each category as; education, financial incentives, generic substitution, advertising approaches, and enforcement.

**Conclusion:**

Various policies should be taken into consideration to encourage successful generic medication prescribing, dispensing, and consumption in both supply and demand-side. Economic, political, socio-cultural, technological, legal, and structural factors could as such accelerate the policies' effect. Studying the experience of successful countries can be helpful for policymakers.

## Introduction

Medicines and medical technologies are considered to be important components of health system building blocks (1). Access to essential medicines in both public and private sectors is a vital part of treatment procedures, particularly, in low-and-middle-income countries (2). The cost of medicines for healthcare systems has significantly increased in the recent years due to the changing pattern of diseases, growing prevalence of non-communicable diseases (NCDs), advances in medical technologies, production of diverse and expensive medicines, aging population, easy access to medications, increase in life expectancy and life quality (3–6). Some countries approximately spend 15–70 percent of their total health expenditures on medicines and 60–90 percent of households' health related out of pocket payments are spent on medicines (7). The share of pharmaceutical expenditures of total health care expenses is different considering the development status of countries. It can be up to 70% of total health care expenditures in lower-middle-income countries compared to up to 17% in higher-income countries (8). For example In Iran, as a developing country, the share of pharmaceutical expenditures are about 30% of total expenditures for healthcare (9). in Canada, US, japan and France, as a developed countries, the share of pharmaceutical expenditures are less than 20% of total expenditures for healthcare (10). It is proved that the costs of medicine are one of the main expenditures of healthcare systems, the second after the healthcare manpower costs around the world (11). Therefore, possible reduction in medicines' cost is one of the everlasting concerns of policy-makers and governments (12). The growing use of generic medicines instead of branded medicines considered to be a key policy to decrease expenditures, improve access to medicines and extend the medical coverage (13, 14). Generic medicines are similar to the branded, except they are nearly 20–90 percent cheaper (15, 16). World health organization (WHO) defines generic medicines as “a pharmaceutical product, usually intended to be interchangeable with the innovator product, which is usually manufactured without a license from the innovator company and marketed after the expiry of the patent or other exclusivity rights” (17). According to food and drug administration (FDA) “A generic medicine is a medication created to be the same as an existing approved brand-name medicine in dosage form, safety, strength, route of administration, quality, and performance characteristics” (18). Generic and branded medicines are the same in active pharmaceutical ingredients (API), effectiveness and benefits. However, the former might be different in its excipients, color, shape, size, and packaging (18).

Governments are well aware that the use of generic medicines might increase the competition among pharmaceutical companies, and in a way, reduce the costs on the pharmaceutical products and healthcare expenditures (13,14). Following the expiration of originator company's medicines patent, generic medicines can be produced. Given bypassing the development and approval process costs, generic drugs, unlike branded medicines can provide the same therapeutic benefits at a lower price(19).

Developed countries applied various policies and regulations to improve the use of generic medicines (20). For example the share of generics in Europe was raised from 42% in 2004 to 49% in 2009 and similarly, in the USA, it increased from 19% in 1984 to 54% in 2005 (21, 22). Generic substitution and generic prescribing are two common strategies for controlling the escalating cost of the medicines (23).

All policies, depending on which group will be affected, generally, can be divided into two fundamental categories: supply-side and demand-side policies. The supply-side policies mostly affect pharmaceutical manufacturers and markets by legislation and policy on market entry and penetration, pricing, intellectual property rights, quality assurance(5, 24), While, demand-side policies relate to prescribers, dispensers, and patients (25).

There are plenty of articles focusing on generic medicine policies (5, 16, 23, 26). Therefore, this study aims to systematically create and present an updated, comprehensive, and summarized overview of the demand-side policies and practices regulating generic medications.

## Methods

**Paper identification**: A comprehensive, though precise, search strategy was used to identify all related articles from around the world in line with the research question from date 1.1.2000 to 12.5.2019. Keywords included “Generic, brand, pharmaceutical, medication, drug, medicine, policy, strateg* and regulation”. We carried out the search in Medline, Scopus, Web of Science, and Embase (appendix table 1). The reference lists of selected articles and general web search engines were also hand searched, to identify other additional relevant studies. The articles were imported into Endnote X7.5.

**Paper selection and extraction**: Following the identification of papers, first all duplicates were deleted, then, two reviewers (MS & EJP) screened the titles and abstracts for relevance, based on the inclusion criteria. The peer-reviewed papers and publications describing policies, regulations and strategies upon the acceptance and adoption of generic medicines by prescribers, dispensers, patients, and healthcare organizations in English language were considered. Letters, editorials, book reviews, conference papers, commentaries, and scientific reports were excluded.

Then, the body of papers were assessed for eligibility by consensus. At this stage using related quality assessment checklists (eg. CASP), the survived papers were assessed in terms of their rigour and methodological capabilities. A data extraction form developed by researchers, including mainly the title/objective of study, the year of publication, study design/setting, policy-related domain for encouraging the use of generic medicine, was used.

All stages are conducted according to the Preferred Reporting Item for Systematic Reviews and Meta-Analyzed (PRISMA).

**Analysis**: The ultimate number of papers were categorized through meta-synthesis, using thematic analysis method (27).

## Results

**Figure 1:**
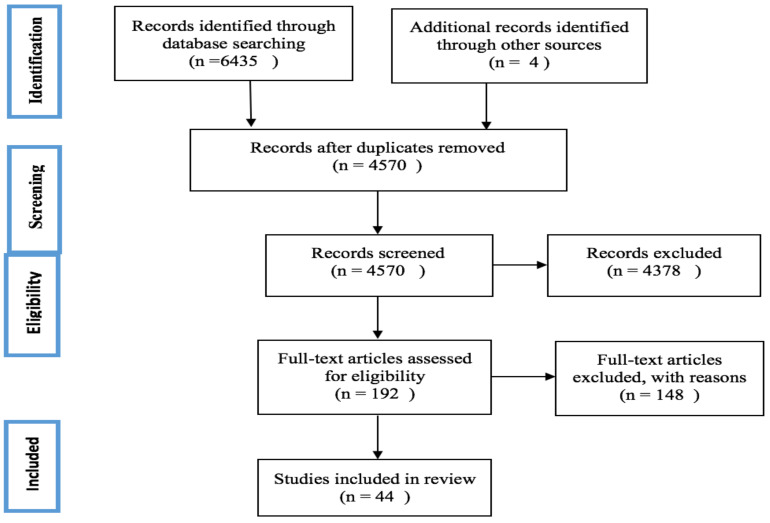
PRISMA flow diagram

A total of 6435 records were overall retrieved from PubMed (n=315), Scopus (n=4323), Web of Science (n=71) and Embase (n=1726) (Figure 1). Ultimately, 44 articles were chosen (appendix table 2), passing all screening procedures, and thematically analyzed. The emerged policies and practices were classified under four key policy-related domains (Table 1).

**Table 1:** Overview of policies and practices encouraging the use of generic medicines

Policy Domain	Sub-domains	Interventions and procedures
**Prescribing**	Education	Educational campaigns, prescribing guidelines, use of emails, official newsletters, mass media, social media, discussion groups (involving doctors, pharmacist, local pharmacotherapeutics discussion between physician and pharmacists (3, 10–13, 16, 23, 24,28–38,43,50)
	Financial incentives	Fixed fee per item, pay for performance and budget cap (16, 23, 24, 28, 33–38, 40–42)
	Enforcement	Forcing physicians by law to write generic name or INN, oblige prescribers to inform patients about the existence of cheaper alternatives (29, 33, 43–46)
	Use of technology	E-prescribing system, mobile applications (5, 16, 19, 23, 24, 31, 32, 35, 38, 43, 58)
	Promotional approaches	Limiting the interactions between physicians and pharmaceutical industries (50, 36)
	Supervisory and feedback actions	Medicine Utilisation review and benchmarks (24, 28)
**Dispensing**	Education	Guidelines, websites, campaigns, newsletters, Proper Education of students in pharmacy school (3, 12, 13, 28, 34)
	Generic substitution	Mandatory, voluntary, forbidden (3, 5, 23, 25, 28, 29, 31–33, 35, 38, 40, 46, 60)
	Financial incentives	Fixed Fee per item, fee-for -performance payment, Regressive margins or regressive markup, Ceilings on the markup of expensive medicines (5, 23, 25, 29, 32–35, 38, 41, 42, 44, 46)
**Patients/Consumers**	Education	Mass media publicity campaign, educational campaigns, information campaigns, anthropomorphic images, Poster and pamphlet, Media campaign in TV, radio and internet (3, 10, 12, 13, 16, 19, 25, 28, 31, 33, 35)
	Financial incentives	Caps, Tiered co-payment, Reference-based pricing system (3, 5, 13, 16, 19, 23, 24, 28, 32, 33, 38, 40, 60)
	Advertising approaches	Prohibition of direct-to-consumer pharmaceutical advertising (DTCPA), Limiting the distribution of free medicine samples (3, 16, 25, 44)
**Healthcare** **organizations**	Education	Guidelines on primary health care problems, leaflets in hospitals and healthcare centers, billboards and the internet (30, 31)
	Enforcement	Mandatory use of generics by hospitals, Mandatory Prescription by INN, Mandatory generic substitution in the inpatient setting (35, 42, 45)
	Financial incentives	Rewards for achieving budget goals and penalties for budget deficits, Fixed fee per item or fee for prescriptions, Pay for performance (p4p), Budget-cap (5, 30, 31, 39, 42)

**Category 1: Prescribing**

Six themes related to the physicians' prescribing practices were identified: education, financial incentives, enforcement, use of technology, promotional approaches, and supervisory and feedback actions.

**Category 2: Dispensing**

As to the dispensing of medications, three themes emerged; including education, generic substitution, and financial incentives.

**Category 3: Patients/consumers**

This category comprised of: education, financial incentives, and advertising approaches

**Category 4: Healthcare organizations**

Three main themes were identified in relation to healthcare organizations: Education, Enforcement, and Financial incentives.

## Discussion

This study set out to provide an overview of policies and practices upon the use of generic medicines in different countries. Various interventions were introduced and adopted to encourage the application of generic medication, discussed under four general domains.

**Prescribing**

**Education**: As indicated in the literature, the majority of physicians are hesitant about prescribing generic medicines, as they are not completely assured of the safety, equivalency, and quality of this type of medication. According to research in Iran, more than 70 percent of physicians declared that they would prescribe these medications if their equivalency was guaranteed. Therefore, it is essential to instruct physicians about the processes behind and involved with producing generic medicines, including the differences between the brand and generic drugs as well as the advantages of such medications. Various strategies are raised to meet this end such as; educational campaigns (as in New Zealand), prescribing guidelines (24,28–33), use of emails, official newsletters, mass media, social media; discussion groups involving doctors, pharmacist, policymakers and manufacturers (11); INN prescribing is encouraged at early stages as medical schools (28,31,34–36), medicine information bulletins (32), academic detailing (e.g. in Belgium) (23, 30,31,37), quality circles for pharmacotherapy (QCPs) (23,37), pharmacotherapeutics discussion group (23), Active feedback, prescribing meetings, prescribing advice to the local general practitioner from community pharmacist, clinical audit (37), government promotional campaigns (31), and local pharmacotherapeutics discussion between physician and pharmacists (24).

**Financial incentives**: This policy can be used in individual (the physicians who are working in private offices) or organizational level (physicians who are working in hospitals or primary health care centers). Organizational physicians are the target of this policy more than physicians who are working in private offices. These incentives can operate in different ways includes: Fixed fee per item, pay for performance, and budget cap that will be described in the category of healthcare organizations (16,23,24,28,31–36,38–42).

**Enforcement**: This concept comprises of two sections. The first one is forcing physicians by law to write generic name or INN (29,33,36,43–46), and the second is to oblige prescribers for informing patients regarding existence of cheaper alternatives (29).

**Use of technology**: The main part of this theme is using of e-prescribing system by physicians (5,16,19,24,32,38,43,47). In this system, medicines are categorized into three tiers. Tier 1 contains low-cost and generic medicines. The second tier includes relatively priced brand-name and third-tier medicines are expensive brand-name medicines with the lowest copayment (48). E-prescribing system can be integrated with decision support systems (DSS) or no (31,35).

**Promotional practices**

*Promotion of generic medicines*: the internet is a good opportunity for manufacturers and regulatory authorities to promote generic medicines in this platform and increase awareness of physicians (36).

*Limiting the interactions between physicians and pharmaceutical industries:* Sometimes physicians may be unable to distinguish between scientific evidence and promotional information (49). According to the evidence, on the other hand, Interactions between physicians and pharmaceutical sales representatives may influence prescribing behavior and probably lead to irrational prescribing of the company's medicine (50). Therefore, some legal limitations are needed to be regulated by governments or medical institutions (51,52).

**Supervisory and feedback actions**

*Medicine Utilization review*: Drug utilization review (DUR) or Drug use evaluation (DUE) is an ongoing, authorized, structured, and systematic quality improvement process, that may provide feedback on the performance of physicians and prescribing patterns in comparison to predetermined criteria or treatment protocols (28, 53). this is a common method of evaluating and refining the appropriateness of medicine prescriptions and plays a key role in helping the healthcare organizations to interpret and improve the prescribing, administration, and use of medicines (53 54).

**Dispensing Education**: Pharmacists' misconception about quality, safety, and efficiency of generic medicines is an important obstacle that prevents their extensive use of them. Ongoing educational interventions can change this misconception (11,55). For example, the National Prescribing Service and Pharmaceutical Society of Australia, continuously provide guidelines and educational materials for community pharmacists through its websites, campaigns, and newsletters (56). Proper Education in pharmacy schools is another strategy that can effectively address the prevailed misconception (57).

**Generic substitution (GS)**: In terms of generic substitution right for dispensers, terms and conditions vary from country to country. GS is mandatory in 11 EU countries, including Sweden, Finland, Italy, and Germany. In 14 countries, it is advised to use GS, including Norway, Ireland, and Poland. As well, the GS is mandatory in 14 US states alternatively, it is forbidden in five countries, including Bulgaria, Austria, and Belgium (3,5,19,23,25,28,29,31–33, 35,38,40,46,58–61). Many studies have shown the efficiency of generic substitution policy and the right of pharmacists to dispense generics, regardless of whether the prescriber wrote the brand name or generic (62–64).

**Financial incentives**: There are various ways to provide financial incentives. Some of them are described in the following:

*Fixed fee per item:* It's a traditional payment method through which dispensers receive a fixed fee per prescription or per medicine if they dispense a generic one (5,25,29,34,35,38,41,42,46,65).

*Pay-for-performance payment*: Which dispensers' income is associate with their performance (e.g. knowledge, consultation, and advising patients to accept generic medicines). In addition to the usual dispensing fee and it is less dependent on the price of medicines (e.g. Belgium and Netherland) (23,66).

*Regressive margins or regressive mark-up:* according to this method, margins, and mark-ups decrease if the price of medicines increases (67). Which the profit of generic and brand medicines is almost close or equal. Therefore, pharmacies are motivated to sell generic medicines. Sweden, Denmark, and France are using this method (5,29,32–34,46,65,68).

*Setting expensive medicines mark-up ceiling:* It contains establishing a markup ceiling for expensive branded medicines. If the price of a medicine exceeds a certain amount, the markup will be lower. For example, in British Colombia State, Canada, the mark-up for medicines with a price of more than 40$ is 5% and for medicines with a price of less than 40$ is 8% (44).

**Patients/consumers**

**Education:** Many studies have shown that misconception is the main reason for the negative attitude of patients about the efficiency and safety of generic medicines. Education and interaction between patients and health care providers (especially physicians and pharmacists) will be effective in changing this misconception (69).

Educational and informational campaigns in television, radio (11,31), internet (23) and social media (11), local newspapers, business journals, consumer magazines, anthropomorphic images (16), Posters and pamphlets (for example in Japan) (25), are practices of encouraging the use of generic medicines in different countries (3,12,19,33,35,38).

**Financial incentives**: There are different policies to influence patients' direct payments (i.e. caps, fixed co-payment, co-insurance, tier co-payment, and ceilings), but just three of them (i.e. caps, tier co-payment, reference-based pricing system) may incentivize patients to select generic medicines (19,59,73). These Policies may overlap in some features.

*Caps:* Determining the ceiling for the number of prescriptions or medicines that are reimbursed in A period (e.g. monthly) (39,58).

*Tiered co-payment:* In this method, according to the costs, effectiveness, and usage, medicines assign to three categories (or even more), and patients should pay more co-payment if they choose to receive medicines that are at higher tiers. Tier 1 is for inexpensive (generic)medicines. The second includes some generics and preferred brand medicines. Expensive and Non-preferred brand medicines are categorized in the third tier (5,16,23,35,38). *Reference-based pricing system:* In this system, patients should pay the difference between prices of medicine and reference prices (announced price by a health insurer). Patients pay these differences, if the price of selected medicine by them, be more than the reference price (3,5,13,23,24,26,32,33,35,40,60).

**Advertising methods**

*Prohibition of direct-to-consumer pharmaceutical advertising (DTCPA):* In most countries (except for the USA, New Zealand, Hong Kong, and Brazil), medicine manufactures are banned from DTCPA (64). The DTCPA affects the patients' and physicians' behavior and causes challenges. Studies have shown that DTCPA increases the market share of branded medicines and, therefore, led into increased costs in the long term, although these effects may not rise in the short term (65).

*Limiting the distribution of free medicine samples:* Studies show that presenting of free branded medicines by physicians leads to physician prescribing habits change and increases prescription of branded (and expensive) medicines. Hence, limitation or prohibition of free samples distribution can be applied as a cost-containment policy (16,44). the cost of free medicine samples is almost half of the marketing cost, so manufacturers compensate these costs through increased prices (66). It is recommended that physicians and pharmacists should prescribe and dispense free medicine samples for poor patients to reduce medicine cost-related nonadherence (CRN) (67).

**Hospitals and primary healthcare centers**

Increasing the proportion of generic medicines in hospitals not only decreases costs but also may affect the consumption of medicines after discharge. After discharge from healthcare centers patients may seek the same medicines that they received during hospitalization, mainly due to their positive mentality (68).

**Education**: *Guidelines* on primary health care problems (30), leaflet**s** in hospitals and healthcare centers, as well as billboards and the internet (31), providing information on therapeutics to primary healthcare physicians by Pharmacists and clinical pharmacologists, are some interventions that they can improve the knowledge of healthcare staff about generic medicines (30).

*Benchmarking primary healthcare physician prescribing:* Benchmarking may play the role of educational intervention if measurable standards and items set for learning (30).

**Enforcement**

*Mandatory use of generics by hospitals:* In 2002, the government of Japan instructed all national hospitals to use generics. As a result, in hospitals with 200 beds or more, the share of generic medicines increased from 0.7% in 2001 to 7.5% in 2003 (42).

*Mandatory Prescription by INN:* As, the names of generic medicines, mostly, reflect the function, nature, effect, and use of medicines, it is too important that prescribers recall more generic medicine names. Therefore, mandatory prescribing by INN (with an e-prescribing system or no) can be an influential option (45, 69).

*Mandatory generic substitution in the inpatient setting:* This policy can be used for patients with lower income and would yield significant cost-saving for hospitals if it can be extended to other settings (i.e. outpatient setting) (35).

**Financial incentives**

*Rewards for achieving budget goals and penalties for budget deficits:* This intervention contains rewards for achieving budget goals and penalties for budget deficits. For example in Catalonia, the Catalan Department of Health, developed some financial incentives for primary healthcare centers (PHC) that the main target areas were the Increase in the prescribing of generic and lower-cost medicines (30).

*Fixed fee per item or fee for prescriptions:* In this way, prescribers incentivize and receive rewards based on the number of prescriptions that they prescribe by INN (e.g. Japan) (6,35,41,42).

*Pay for performance (p4p):* This payment model offers financial incentives to physicians for meeting certain performance measures, regarding the prescription of generic medicines (16,31,33). For example, France started the CAPI scheme from 2009. this scheme has 16 measure that 5 of them are about prescribing of generic forms of antibiotics, proton pump inhibitors, statins, antihypertensive medicines, and antidepressants (67).

*Budget-cap:* This model is more common among primary healthcare physicians and known as Pharmaceutical prescription budgets (38), budget target or medicine budgets (23, 35), fund-holding schemes (UK) and physician budgets (16,24,28,34,40). In this way, a specified budget is allocated to prescribers or HCOs and they should render services considering cap (5). Saving achieved by the physicians can be used for training (35).

The use of generic medicines could increase the access of people to medicines, improve medication adherence, and in a way decrease the healthcare expenditures. Countries are advised to apply different approaches (enforcement, encouragement, support) for prescribing, dispensing, and consumption in both supply-side and demand-side related policies. Therefore, consideration of economic, political, socio-cultural, technological, legal, and structural factors might prepare the ground for more relevant policies. Insights and experiences of successful countries are expected to assist policy-makers and managers in various settings to manage their medication practices effectively.

This paper provides a comprehensive package of driving policies and practices for promoting the utilization of generic medication. Results are well-explored and categorized. However, extending the period under investigation might provide more evidence.
